# FLP/*FRT* Recombination from Yeast: Application of a Two Gene Cassette Scheme as an Inducible System in Plants

**DOI:** 10.3390/s100908526

**Published:** 2010-09-13

**Authors:** Murali R. Rao, Hong S. Moon, Tobias M. H. Schenk, Dirk Becker, Mitra Mazarei, C. Neal Stewart

**Affiliations:** 1 Department of Plant Sciences, University of Tennessee, Knoxville, TN 37996, USA; E-Mails: mraghave@utk.edu (M.R.R.); hmoon@utk.edu (H.S.M.); nealstewart@utk.edu (C.N.S.); 2 Biocentre Klein Flottbek, Developmental Biology and Biotechnology, University of Hamburg, Hamburg, Germany; E-Mails: t.schenk@botanik.uni-hamburg.de (T.M.H.S.); becker@botanik.uni-hamburg.de (D.B.)

**Keywords:** FLP/*FRT*, site-specific recombination, heat shock, GUS reporter, tobacco, *Arabidopsis*, phytosensing

## Abstract

Phytosensors are plants that are genetically engineered for sensing and reporting the presence of a specific contaminant, including agriculturally important biological agents. Phytosensors are constructed by transforming plants to contain specific biotic- or abiotic-inducible promoters fused to a reporter gene. When such transgenic plants encounter the target biotic or abiotic agent, the specific inducible promoter is triggered and subsequently drives the expression of the reporter gene, which produces a signal for detection. However, several systems lack robustness, rapid induction and promoter strength. Here, we tested the FLP/*FRT* recombination system in a construct containing a two gene cassette organization and examined its potential in transgenic *Arabidopsis* and tobacco plants using a β-glucuronidase (GUS) reporter. In this model system, a heat-shock inducible promoter was employed to control the expression of the FLP recombinase gene. Upon heat induction and subsequent active FLP-mediated excision event, the GUS gene was placed in close proximity to the 35S promoter resulting in an active GUS reporter expression. Our results demonstrate that the two gene cassette scheme of inducible FLP/*FRT* recombination system is functional in tobacco and *Arabidopsis*, providing additional insights into its possible application in phytosensing such as creating strong readout capabilities.

## 1. Introduction

The use of real-time *in vivo* systems to detect the presence of chemical contaminants or agriculturally important biotic/abiotic stressors provides an imperative first line of protection for environmental- and plant health [1–5]. Sentinels utilizing plants genetically engineered to produce a specific signal upon induction by an agent is envisioned as one phytosensor platform [6–8]. Engineered phytosensors could contain contaminant- or biotic/abiotic-inducible promoters driving the expression of a reporter gene, whose presence is, in turn, detected by visual observation or some device [9]. When such transgenic plants encounter the agent, the specific inducible promoters are triggered and subsequently drive the expression of the reporter gene producing a signal for detection.

Plant sentinels, or ‘phytosensors’, potentially have tremendous utility as wide-area detectors. The use of an inducible promoter is a desirable feature when contemplating its use for the design of a phytosensing system. However, the complexity of the expression of many plant promoters is inherent to the fact that they contain several *cis*-regulatory elements, each of which can be activated or repressed by more than one agent. To avoid this limitation, synthetic promoters can be designed based on selected *cis*-regulatory elements [9]. Yet, from our previous results [10,11], one major problem associated with employing either native or synthetic inducible promoters directly fused to a reporter gene is the lack of sufficient expression of the reporter, *i.e.*, the inducible promoter is not strong enough to produce robust signals upon full induction—the most fundamental feature that is expected to result when induced by the agent in a phytosensing system. Although increasing basal level of the reporter expression by addition of enhancer elements in the synthetic inducible promoter constructs was achievable [11], detection of a false reporter signal from high-level background of reporter expression prior to the real-time induction in response to the agent would be considered as a potential drawback.

To address the problem associated with the inducible promoters directly fused to a reporter in phytosensing systems, we expanded the phytosensing platforms to include a site-specific recombination strategy. Site-specific recombination involves enzyme mediated rearrangement of DNA fragments that do not possess a high degree of homology [12,13]. Conservative site-specific recombination (CSSR) and transposition are two classes of site-specific recombination [13,14]. CSSR involves exchange or recombination at highly specific regions within short stretches (recombination sites) of identical sequences in the participating DNA fragments, while transposition does not require any homology between the recombination sites [13]. CSSR can result in different DNA rearrangements depending on the relative orientation of the recombination sites, in *cis* and oriented in the same direction results in deletion of the DNA fragment contained within the two sites, while recombination sites in *cis* and in the opposite orientation results in inversion of the DNA fragment [12,13]. On the other hand, having recombination sites in *trans* on two linear DNA molecules results in exchange of DNA fragments. If one of the DNA molecules involved in the reaction is circular, recombination results in a cointegration event, but this event is kinetically less favorable and less likely to occur [12]. There are several CSSR systems identified and shown to be functional in higher eukaryotes. These simple and efficient CSSR systems can have a wide variety of applications in plant biotechnology. Some of these applications include excision of selectable marker genes from transgenic plants, excision of redundant copies of transgene in crop plants to reduce the extensive screening required to obtain single-copy transgenic lines, and site-specific integration of transgenes [15–30].

**Figure 1.**
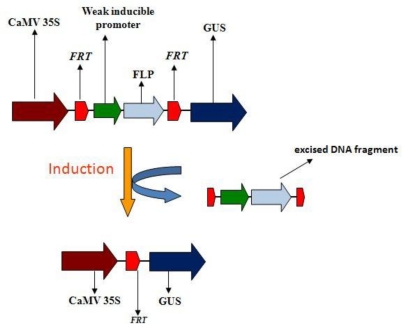
Schematic representation of the FLP/*FRT* site-specific recombination system in phytosensing. Upon induction, the FLP recombinase protein produced will recognize the FRT sites and the region between these two FRT recognition sites will be excised and the reporter gene GUS is brought under the influence of the CaMV 35S promoter resulting in plant-wide expression of GUS. FLP—flipping DNA recombinase, FRT—FLP recombination target; GUS—β-glucuronidase.

Site-specific recombination systems have been used primarily for the deletion of transgenes resulting in antibiotic-free transgenic plants and/or for direct integration of transgenes into plant genomes. In this study, we propose possible application of this system for phytosensing purposes. We employed one of the well-characterized site-specific recombination systems—the FLP/*FRT* from *Saccharomyces cerevisiae*, where FLP (flipping DNA) is the recombinase which recognizes *FRT* (FLP recombination target) sites [12,21]. We used an inducible heat-shock promoter to produce a system in phytosensing using both tobacco and *Arabidopsis* as phytosensor models. We used a two gene cassette system in which the inducible heat-shock promoter, instead of controlling the expression of the GUS reporter gene directly, regulates the expression of the FLP recombinase gene, so that once induced by heat, the recombination would result in the excision of the DNA fragment between the *FRT* recognition sites, then placing the CaMV 35S promoter in close proximity of the reporter gene. This (in case of a weak inducible promoter driving the recombination) would lead to efficient detection of the signal (Figure 1). Our results provide additional insights into the possible application of site-specific recombination system as a tool for the reporter signal in phytosensing.

## 2. Experimental Section

### 2.1. Vector construction

**Figure 2.**
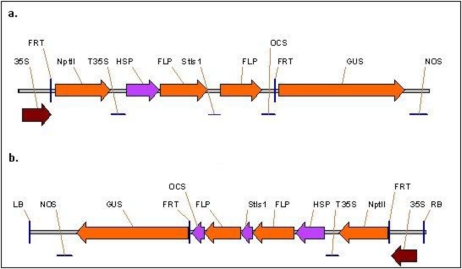
Schematic diagram of the biolistic and binary vectors. **(a)** The source plasmid of the recombination cassette. **(b)** Region between the left border (LB) and right border (RB) in the binary vector pBIN-HSP-FLP-GUS-Hyg containing the recombination cassette. 35S—CaMV 35S promoter, FRT—FLP recombination target, NptII—kanamycin resistance gene, T35S—CaMV 35S terminator, HSP—heat shock promoter, FLP—flipping DNA recombinase, Stls1—intron from potato, OCS—octopine synthase terminator, GUS—β-glucuronidase, NOS—nopaline synthase terminator.

A biolistic vector, useful for plant transformation via biolistic particle delivery system, containing the FLP-*FRT* recombination cassette with GUS as the reporter gene (Figure 2a) [16] was used as template to PCR amplify the FLP-*FRT* recombination cassette. The forward and reverse primers used were: 5′-TATCATCACGTAGTGCATTTCCCCGAAAAGTGCCAC-3′ and 5′-TATCATCACGTAGTGCTTTTTACTAGAGGCCTTGGG-3′, respectively. The PCR amplification was performed with *pfu* Ultra II Fusion HS DNA polymerase (Stratagene, La Jolla, CA, USA) and the conditions were 95 °C for 2 min followed by 30 cycles of 95 °C for 30 s, 60.2 °C for 1 min, and 72 °C for 8 min and then was followed by a final extension at 72 °C for 10 min. The PCR product was inserted into pBIN19 binary vector between the left and the right borders of the T-DNA using *Dra III* restriction enzyme (NEB, Ipswich, MA, USA) (Figure 2b). The recombination cassette comprises a kanamycin resistance gene (*nptII*) and a FLP recombinase gene driven by a soybean heat-shock promoter (Gmhsp 17.5-E) between the two FRT sites. *Stls1* is an intron from potato inserted within the FLP gene. The CaMV 35S promoter drives expression of the *nptII* gene before recombination whereas it drives the GUS gene after recombination when the fragment between the FRT sites is excised. As controls we used binary vectors pBI-35S-GUS (GUS driven by the CaMV 35S promoter), and pBI-HSP-GUS (GUS driven by soybean heat-shock promoter Gmhsp 17.5-E). None of the control vectors contained recombination components.

### 2.2. Plant transformation

*Arabidopsis thaliana* (ecotype Columbia) and *Nicotiana tabacum* (cv Xanthi) plants were transformed with each vector construct using *Agrobacterium tumefaciens* strain GV3850. *Arabidopsis* transformation was performed by the floral dip method [31] and tobacco transformation by the leaf disc transformation method [32]

### 2.3. Heat-induction experiments

Transgenic tobacco and *Arabidopsis* lines (T2 generation) were grown at 22 °C with photoperiod of 16 h. Tobacco and *Arabidopsis* plants were heat-shocked at 42 °C for 6 h and then returned to growth chambers to recover for 24 h. The plants were then analyzed for GUS expression.

### 2.4. GUS assay

GUS expression analyses were performed by subjecting the plants to histochemical assays using GUS staining solution containing the substrate 5-bromo-4-chloro-3-indolylglucuronide (X-Gluc) as described in [33]. Leaf samples were immersed in GUS staining solution and vacuum infiltrated for 10 min following overnight incubation at 37 °C. After GUS staining, tissues were cleared by replacing the GUS solution with 70% ethanol and then examined for GUS expression.

### 2.5. PCR assay

Genomic DNA was extracted using Qiagen DNeasy plant mini kit (Qiagen Inc., Valencia, CA, USA) and PCR was performed using *Takara EX Taq* (Takara Bio Company, Madison, WI, USA) to verify excision of DNA fragment post heat-induction. The PCR conditions were 94 °C for 2 min followed by 40 cycles of 94 °C for 30 s, 64.2 °C for 30 s, and 72 °C for 4 min 30 s and then was followed by a final extension at 72 °C for 10 min. PCR products were separated on a 1.2% agarose gel with ethidium bromide in 1X TAE buffer.

## 3. Results and Discussion

**Figure 3.**
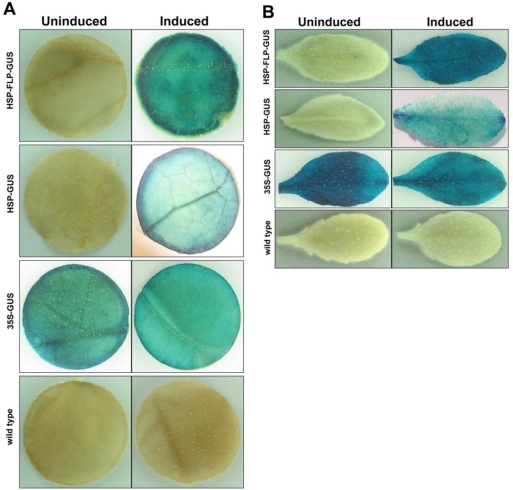
Histochemical analysis of GUS expression in leaf tissues of tobacco **(A)** and *Arabidopsis* **(B)** plants exposed to heat-shock at 42 °C for 6 h. HSP-FLP-GUS (transgenic containing FLP/FRT recombination system); HSP-GUS (transgenic control: heat-shock promoter driving GUS expression); 35S-GUS (transgenic control: CaMV 35S promoter driving GUS expression); wild type (non-transgenic control).

A two gene cassette scheme of an inducible FLP/*FRT* recombination system was examined in tobacco and *Arabidopsis* plants. A plant expression vector containing the inducible FLP/*FRT* recombination system was constructed and the utility of the system was examined by employing a heat-shock promoter for induction of the system. In this two gene cassette system, the FLP recombinase gene was driven by soybean heat-shock inducible promoter where the expression of the FLP gene upon heat-induction would lead to excision of the DNA fragment between the *FRT* sites. This subsequent recombination event would place the CaMV 35S promoter within close proximity with the GUS gene thereby resulting in an active expression of the GUS reporter (Figure 2). Histochemical analysis of GUS activity for tobacco (Figure 3A) and *Arabidopsis* (Figure 3B) showed that the heat-shock treatment successfully excised the DNA fragment between the *FRT* recognition sites by driving the expression of the FLP recombinase gene further resulting in the CaMV 35S promoter driven expression of the GUS reporter gene in a predictable fashion. The expression of GUS in the transgenic plants that contained the recombination system was comparable to the positive control where the GUS expression was driven by CaMV 35S in a direct fusion system. A total of six lines out of 11 independent tobacco lines and six lines out of 14 independent *Arabidopsis* lines carrying the recombination system exhibited GUS activity upon heat induction. These plants displayed a uniform pattern of GUS expression with a range of medium to high GUS activity relative to the control (data not shown). We also observed GUS activity in a few of the uninduced counterparts in both tobacco and *Arabidopsis* plants. The leakiness of heat-shock promoters has been also observed in other studies [34,35].

**Figure 4.**
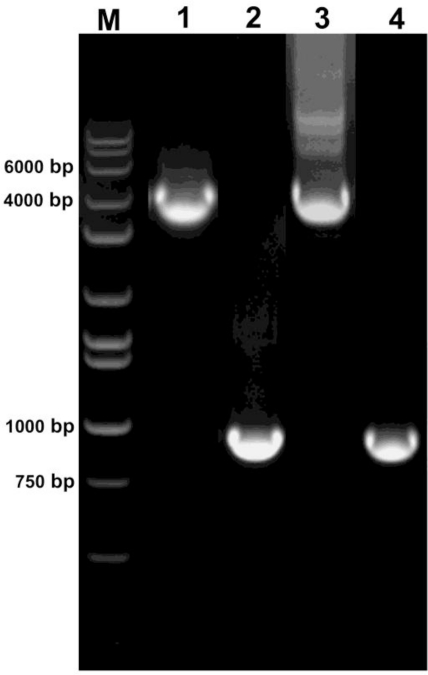
PCR results (representative) from heat-induced and un-induced tobacco and *Arabidopsis* plants. PCR fragments of size 4,165 bp and 931 bp is expected from un-induced and induced plants, respectively. Similar results were obtained by PCR analysis of several independent lines of tobacco and *Arabidopsis*. M—HI-LO DNA marker, 1—Tobacco un-induced, 2—Tobacco induced, 3—*Arabidopsis* un-induced, 4—*Arabidopsis* induced.

For further verification, the recombination event upon heat induction was analyzed by PCR. A successful recombination event in a heat-treated plant would result in a 931 bp PCR fragment whereas in an untreated plant would result in a 4,165 bp PCR fragment. As shown in Figure 4, the 931 bp PCR fragment was amplified for the heat-induced plants confirming the excision of the DNA fragment between the two FRT sites whereas the 4,165 bp PCR fragment was amplified for the untreated plants. Induction was analyzed by PCR in all the independent tobacco and *Arabidopsis* lines exhibited GUS activity upon heat induction and similar results were obtained (data not shown).

Taken together, our results demonstrate that this two gene cassette organization of the inducible FLP/*FRT* recombination system is functional in tobacco and *Arabidopsis* plants, suggesting its possible application for reporter signal manipulation in phytosensing.

## 4. Conclusions

Phytosensors could serve as a valuable and a cost-effective method for sensing environmental contaminants in real time. An inherent problem associated with this application is the inadequate level of reporter signal for detection, which depends, at least in large part, on the strength of the inducible promoters involved in phytosensing. In this study, we demonstrated that a two gene cassette scheme of the inducible FLP/*FRT* recombination system is functional in tobacco and *Arabidopsis*. This system can be used as a potential tool for the reporter gene signal from phytosensor plants when the inducible promoters may provide sensing capabilities but are not sufficiently strong to produce robust signals needed for phytosensing.
